# Demand for Health Information on COVID-19 among Vietnamese

**DOI:** 10.3390/ijerph17124377

**Published:** 2020-06-18

**Authors:** Huong Thi Le, Diep Ngoc Nguyen, Ahmed Sam Beydoun, Xuan Thi Thanh Le, Thao Thanh Nguyen, Quan Thi Pham, Nhung Thi Kim Ta, Quynh Thi Nguyen, Anh Ngoc Nguyen, Men Thi Hoang, Linh Gia Vu, Bach Xuan Tran, Carl A. Latkin, Cyrus S.H. Ho, Roger C.M. Ho

**Affiliations:** 1 Institute for Preventive Medicine and Public Health, Hanoi Medical University, Hanoi 100000, Vietnam; lethihuong@hmu.edu.vn (H.T.L.); lethithanhxuan@hmu.edu.vn (X.T.T.L.); nguyenthanhthao@hmu.edu.vn (T.T.N.); quanphamhmu@gmail.com (Q.T.P.); tathikimnhung.hmu@gmail.com (N.T.K.T.); nguyenthiquynhhmu@gmail.com (Q.T.N.); anhnn@hmu.edu.vn (A.N.N.); bach.ipmph2@gmail.com (B.X.T.); 2 Institute for Global Health Innovations, Duy Tan University, Da Nang 550000, Vietnam; nguyenngocdiep7@duytan.edu.vn; 3 Faculty of Pharmacy, Duy Tan University, Da Nang 550000, Vietnam; 4 College of Medicine, University of Cincinnati, Cincinnati, OH 45221, USA; a.sambeydoun@gmail.com; 5 Center of Excellence in Evidence-based Medicine, Nguyen Tat Thanh University, Ho Chi Minh City 700000, Vietnam; linh.coentt@gmail.com; 6 Bloomberg School of Public Health, Johns Hopkins University, Baltimore, MD 21205, USA; carl.latkin@jhu.edu; 7 Department of Psychological Medicine, National University Hospital, Singapore 119074, Singapore; cyrushosh@gmail.com; 8 Department of Psychological Medicine, Yong Loo Lin School of Medicine, National University of Singapore, Singapore 119228, Singapore; pcmrhcm@nus.edu.sg; 9 Institute for Health Innovation and Technology (iHealthtech), National University of Singapore, Singapore 119077, Singapore

**Keywords:** COVID-19, health information, news on the pandemic, Vietnam, risk communication

## Abstract

Due to the rapid spread of coronavirus, Vietnam introduced its first national partial lockdown on April 1st, 2020. The public relied on online sources, whether through official websites or phone-based applications, to acquire up-to-date health information, provide accurate instructions, and limit misinformation. This study aims to provide insight regarding the current level of awareness of the pandemic, and to identify associated factors in Vietnamese participants to recommend necessary interventions. A cross-sectional study was conducted using a web-based survey during the first week of the lockdown period. There were 341 observations collected using a snowball sampling technique. A Tobit multivariable regression model was used to identify factors associated with the demand for each category of health information. The most requested information was the latest updated news on the epidemic, followed by information about disease symptoms and updated news on the outbreak. The prevalence of diverse socioeconomic, demographic, and ethnic factors in Vietnam requires consideration of the specific health information needs of unique groups. Identifying group-specific demands would be helpful to provide proper information to fulfill each population group’s needs.

## 1. Introduction

With the outbreak of the novel coronavirus, which was first identified in Wuhan, China, a pandemic was officially announced by the World Health Organization (WHO) on March 11th 2020, with the name COVID-19 [1]. The high rate of transmission, dangerous complications, and lack of specific treatments make this virus a danger to public health. Since the outbreak of the new pandemic, the rapid increase in the number of confirmed cases and subsequent deaths across the globe has raised the demand for communication-based interventions [2,3]. Due to the isolation and quarantine measures, as well as nonpharmaceutical measures such as social distancing, the most common method for the public to follow and stay updated on health information is through official websites, such as WHO and governmental portals, and specific phone-based applications [4,5,6]. Diversifying health information to meet the demands of various socioeconomic and demographic groups requires not only scientific and academic information, but also reliable instructions for the general population in terms of how to prevent the transmission of coronavirus. A lack of knowledge about the pandemic, out-of-date information, misinformation, and fake news increase the risk of serious consequences [7,8,9]. Due to the nature of emerging infectious diseases, which can lead to fears spreading in the communities, the fight against this pandemic is also the fight against an info-epidemic, especially in developing countries where network securities continue to face challenges [10,11,12].

Vietnam is in the low and middle income countries (LMIC) group with relatively high population density (estimated at 308 people/km^2^), with about 66% of the population accessing internet in 2019, and a healthcare system that requires improvement in several aspects per WHO recommendations [13,14,15,16]; therefore, the residents of Vietnam face a high risk while responding to the COVID-19 pandemic. The high demand for proper health information during this momentous period is a common concern in public health. Recent research on COVID-19 in Hong Kong has illustrated the importance for residents to be equipped with knowledge, attitude, and practices (KAP) for prevention and protection from coronavirus [17]. Most countries provide similar types of information through governmental portals, and Vietnam is not an exception. The awareness of the community regarding symptoms and prevention (e.g., stay at home), is enhanced through key messages that are widely transmitted and seen on mass media, websites, mobile applications, and automatic SMS notifications that deliver information directly and effectively. The efficacy of these channels has been illustrated in previous research on the SARS pandemic [18]. In addition, these methods and warnings increase the vigilance of the whole community regarding the emergency of this pandemic and allow for the self-assessment of the risk from COVID-19 [19], as was observed with previous public health issues such as HIV, Ebola, and SARS [20,21,22]. 

The prevalence of diverse socioeconomic and ethnic factors in Vietnam [23,24] requires consideration of the precise health information needs of each specific population to respond to not only COVID-19, but also other public health problems. A multichannel risk communication program should be considered to provide health information for the overall population. There have been a few studies in Vietnam outlining evidence-based demand for health information [25]; however, no previous research has been carried out during an epidemic and especially during a historic national lockdown. The purpose of this study is to provide insight regarding the current level of awareness of the pandemic and to identify associated factors in Vietnamese participants to recommend necessary interventions.

## 2. Materials and Methods 

### 2.1. Study Design and Sampling Methods

A cross-sectional study was conducted using a web-based survey. This survey was divided into five independent blocks for five different research topics regarding the COVID-19 pandemic. This analysis was conducted within one week (from 6 to 12 April 2020) of national lockdown, enacted on 1 April 2020 due to the COVID-19 pandemic in Vietnam. The government highly recommended Vietnamese residents to stay at home and practice nonpharmaceutical measures (including travel bans and restrictions, contact reductions, and social distancing) in order to reduce the transmission of coronavirus in the community.

Respondents who met the following inclusion criteria took part in the survey: (1) Being at least 18 years old and agreeing to take part in the research by approving the online informed consent; (2) Having access to the web-based survey; and (3) Having ability to read and respond to the questionnaire in Vietnamese. Respondents did not receive any incentives for taking part in this survey to ensure that the same person did not answer the survey more than once, and there was no time limit to complete this survey.

A total of 341 observations (including from hospital and medical university staff, medical students, teachers, officers, and other community members) were collected for the sample within one week.

### 2.2. Variables

#### 2.2.1. Demographic Characteristics

The respondents reported gender, age, marital status, ethnic group, religion, number of living children, educational level, and living areas.

#### 2.2.2. Working-Related Characteristics

Working-related characteristics include job title and current work status.

#### 2.2.3. COVID-Related Information

The respondents were asked to select all types of health-related information on COVID-19 without any priority that they would like to receive. These types of information were decided upon based on previous research on severe acute respiratory syndrome (SARS), Ebola, and recent studies on COVID-19 [26,27,28].

### 2.3. Data Sources

Respondents were recruited using the snowball sampling technique. Hanoi Medical University set up a core group, including 15 selected people, in the recruitment process. The questionnaire was initially sent to this core group. This core group had a higher likelihood of knowing others through the network of medical students (including both former and current ones) and staff in different medical universities in Vietnam. Groups to reflect the diversity of study subjects, including age, gender, and occupation, were identified. The core group sent the link to access questionnaires to their closed contacts once through email, Facebook, or Zalo applications in their computer/smartphone. Those who were next involved in the study received instructions to invite other Vietnamese residents to participate in the survey. Our respondents included health care workers in hospitals, health care centers, staff and students at medical universities, and family members and relatives throughout all 63 provinces of Vietnam.

### 2.4. Data Analysis

STATA 15.0 software was used to analyze the data. We utilized exploratory factor analysis (EFA) to examine the construct validity of the questionnaire. The principal component analysis was used to define factors with a threshold of an eigenvalue of 0.9 by scree test, where the curve was flattened. An orthogonal varimax rotation with Kaiser normalization was applied to explore the scale of items and increase the interpretability of study results. The cut-off point for factor loading was defined at a value of 0.49. We measured the internal consistency of each factor by Cronbach’s alpha. 

Descriptive statistics were used to examine characteristic data, including frequency, percent, mean, and standard deviation. Inferential statistics were applied to perform the comparison between two subject groups (people whose income decreased due to the impact of COVID-19 and whose did not), by T-test or Mann–Whitney test for quantitative variables and by the Fisher-exact test or Chi-squared test for qualitative variables. A Tobit multivariable regression model was used to identify factors associated with the demand for each category of health information. To achieve reduced models, stepwise forward selection strategies were utilized with a log-likelihood ratio test at a *p*-value of 0.2. Statistical significance was defined at a *p*-value of less than 0.05.

### 2.5. Ethical Consideration

The Review Committee at Hanoi Medical University approved the research on 28 March 2020. Information on the purpose of research and informed consent was available on the online web-based survey for participants to examine prior to deciding on participating. Participation was voluntary, and anonymity was assured. Respondents could decline to participate or withdraw from the online survey at any time.

## 3. Results

The response rate of this block was 100%. The socioeconomic characteristics of the participants are summarized in Table 1. Women accounted for 65.7% of the participants. Most participants lived in the Northern region of Vietnam (76.8%) and reported having a religious affiliation (85.0%). About three-fourths of respondents’ family sizes ranged from three to five people. The most common occupation was students (24.6%), and the most common occupation status was tenure. Mean age of respondents was 33.7 ± 10.8, and the mean number of children was 1.1 ± 1.0. There was a no significant statistical difference between respondents whose income was and was not affected by COVID in socioeconomic characteristics

Among the respondents, the most demanded information was updated news on the pandemic (76.0%), followed by information about disease symptoms (63.9%) and updated news on the outbreak (61.0%), as shown in Table 2. The least demanded information was “Notices when traveling” (18.5%). By applying exploratory factor analysis, the study identified three domains in regard to the demand for health information: (a) Updated information on disease and treatment; (b) Transmission mechanism and specific notices; (c) Epidemiology of symptoms, treatment, and prevention. The Cronbach’s alpha of each factor was 0.79, 0.75, 0.77, respectively. On band score from 0 to 1, the mean score of domain a was 0.6 ± 0.4, and the scores of domains b and c were 0.5 ± 0.4 and 0.6 ± 0.4, respectively.

Table 3 reveals the factors associated with the demand for health information on COVID-19 of the participants. Respondents aged 35–44 years old were less likely to demand for COVID-19 “Transmission mechanism and specific notices” and “Epidemiology of symptoms, treatment, and prevention” (Coef. −0.20; 95% CI −0.35 to −0.05 and Coef. −0.45; 95% CI −0.73 to −0.18, respectively). Compared to people who had a high school education level or below, those who had a university/college education level were more likely to request “Updated information on disease and treatment” (Coef. 1.18; 95% CI 0.98 to 1.42). Professional educators and white-collar workers were more likely to want more information about “Epidemiology of symptoms, treatment, and prevention” in comparison to health workers (Coef. 0.63; 95% CI 0.27 to 0.99 and Coef. 0.44; 95% CI 0.11 to 0.78, respectively). Respondents who had limited term full-time employment contracts were more likely to want more information about “Transmission mechanism and specific notices” and “Epidemiology of symptoms, treatment, and prevention” in comparison to tenures.

## 4. Discussion

The findings of this study illustrate that “Updated information on disease and treatment”, “Transmission mechanism and specific notices”, and “Epidemiology of symptoms, treatment, and prevention” were the leading requests for the type of health information about COVID-19 during the national lockdown in Vietnam. However, information about notices when traveling was not in high demand at the time the survey was conducted. 

Our results are similar to other studies. At the outbreak of the COVID-19 pandemic, more than 90% of Chinese respondents desired additional information about COVID-19, including the route of transmission, the availability of medicines/vaccines, travel advice, overseas experience in handling COVID-19, the number of infected cases and locations, advice on prevention of the COVID-19, and symptoms of COVID-19 infection [29]. One month after the outbreak, high satisfaction with health information was significantly associated with lower psychological impact, stress, anxiety, or depression scores [30].

Of the respondent subgroups, postgraduates were likely to have lower demand for “Epidemiology of symptoms, treatment, and prevention” in comparison with the lower educational level group. A possible explanation of this result is that mass media, together with other information channels, provided information on coronavirus for several months prior to the conduction of the survey, and those with a higher educational level and concerns about COVID-19 might have already sought and obtained this information. This finding is consistent with results of a recent study in Hubei, China, which was implemented immediately a week after the lockdown, and indicated that knowledge score about COVID of the master’s degree and above group was relatively higher than the middle school and below group (11.2 vs. 9.7 out of 12) [31]. Similarly, another study in China related to the A/H1N1 pandemic found that the college and above educated group had higher knowledge regarding transmission than those with primary middle school education (66.9% vs. 38% and 55.6%, respectively) [32]. These results further confirm the association between education level and health information seeking behaviors among participants. Another possible explanation is that postgraduates are more likely to have a stable occupation and/or savings, which allows them to weather the pandemic for longer periods with fewer concerns since higher educational levels are associated with job stability and higher income [33,34]. In contrast, the pandemic, in general, and the nationwide lockdown policy, in particular, have a greater impact on the income of low education level populations due to lower job stability and savings. Therefore, these populations might be more motivated to find more sources of information on this issue. The differential impact of public health crises on groups with lower socioeconomic and educational status is common. For example, the rate of acquiring HIV was positively associated with lower education attainment [35] among a population in a province in Canada. Lower education groups have additional economic burdens to overcome public health problems, such as sexually transmitted diseases, pneumonia, and hepatitis B, in both developed countries, such as the USA and Europe [36,37], and developing countries with limited healthcare resources [38,39]. 

Likewise, the results also indicated that the younger age group (below 25 years old) have higher demand for health information on “Transmission mechanism and specific notices” and “Epidemiology of symptoms, treatment, and prevention” compared to the 35–44 years old group. There are several possible explanations to consider for these results. Young people might have more tools and means to access health information, including governmental, healthcare, and personal websites/blogs, mass media, social media, SMS, and phone-based applications [40,41,42]. This increased interface results in more awareness about the risks and hazards caused by public health crises and leads to increased demand for health information [43]. This finding might also be partly be explained by the fact that the middle-age population had higher number of dependents and higher financial risks [44]. Due to having a greater number of dependents (such as children, older family members with health problems), people might have invested time and sought out COVID-related health information prior to the pandemic period. 

The results of this study also provide evidence that health workers had lower demand for “Epidemiology of symptoms, treatment, and prevention” compared to all other occupations. For healthcare professions, they demand more information on first-hand medical information on the outbreak and more training on personal protective equipment and infection control measures during the COVID-19 pandemic [45]. The nonhealthcare workforce request information on personal and organization prevention strategies against COVID-19 when they return to work after lockdown [45]. Epidemiology is an integral part of the formal training that medical students and health workers undergo in the vast majority of countries [46,47]. In addition, the WHO has published a document on basic epidemiology in many languages in order to increase epidemiological knowledge in the community [48]. Healthcare workers play a vital role in the community because of their skillfulness and advanced health literacy. A systematic review study in sub-Saharan Africa demonstrated the contributions of health workers in HIV care among various community settings [49]. Healthcare workers are also the main human resource on the front lines and face the highest risks of daily contact potential to COVID patients during the pandemic. Therefore, information regarding symptoms, treatment, and prevention of COVID-19 needs to be delivered to them in a direct and accurate manner as emphasized in the guidance from the WHO for health workers [50]. At the same time, the United Nations has made special documents available for health workers worldwide to effectively participate in the fight against coronavirus [51]. In addition, many governments have published detailed reports and documents for those who work in healthcare to better respond to COVID-19. 

There are several implications from the findings of this study. The diversity in the demands of health information between different groups, which are based on multiple socioeconomic and educational factors, suggests the need for multilevel health communication with specific targeted programs for different groups. For example, companies and unions could design different training programs for employees by considering their specific educational levels. Governments and healthcare groups should also take advantage of the many organizations, such as the Vietnam Women’s Union, Ho Chi Minh Communist Youth Union, and student associations in schools and universities, that have previously shown their effectiveness in disseminating information and connecting people [52,53,54]. The Vietnamese government has been working to gain the trust of its residents through providing detailed and essential health information. To reduce the dangers of misinformation, they have imposed penalties against individuals who perpetuated fake news and misinformation about the COVID-19 pandemic. Nearly 700 violations have been investigated and individuals have been charged with penalties, both criminal penalties (maximum seven years of prison) and administrative sanctions (maximum 20 million VND, equivalent to 853 USD). In addition to stopping the spread of misinformation, a multidisciplinary approach is needed to deliver applicable and valid information to each individual/group by making use of online human resources, especially in these extensive social distancing circumstances.

Our study has several limitations. First, using the snowball sampling method might result in a less representative study population and decrease the generalizability of the study. Information on the older age group, which has been heavily impacted by the virus in terms of death, was relatively small because of the difficulty of identifying their demand for health information. Secondly, self-reported data collection is susceptible to recall bias, and the nature of cross-sectional study limits the possibility to identify causal relationships. Finally, the online survey is limited because the results cannot be extrapolated to populations that do not have access to the Internet; however, at the time of national lockdown, it was one of the most effective and fastest channels to collect information. Another minor limitation was that there was no significant statistical difference found between respondents whose income was and was not affected by COVID due to the limited sample size of 341 respondents who had similar socioeconomic characteristics. However, the quick response of these respondents helped us address the research question of this study in a relatively short time with a well-designed questionnaire. There are still many unanswered questions about the dissemination of health information, both before and after the national lockdown, that should be addressed to have a comprehensive assessment of the real demands of Vietnam’s diverse population groups.

## 5. Conclusions

The prevalence of diverse socioeconomic, demographic, and ethnic factors in Vietnam requires consideration of the precise health information needs of specific groups. Identifying group-specific demands would be helpful to provide proper information to fulfill each population group’s needs. The Vietnamese government’s approach to gaining the trust of its residents has been through providing essential health information and imposing penalties against individuals who perpetuate fake news and misinformation about the COVID-19 pandemic. However, this health information should be tailored to the needs of specific groups.

## Figures and Tables

**Table 1:** Socioeconomic characteristics of respondents.

Characteristics	Income Decreased due to the Impact of COVID-19				Total		*p*-Value	
	Yes		No					
	*n*	%	*n*	%	*n*	%		
Total	228	66.9	113	33.1	341	100.0		
Gender								
Male	71	31.1	46	40.7	117	34.3	0.08	
Female	157	68.9	67	59.3	224	65.7		
Current living location								
Northern Vietnam	172	75.4	90	79.7	262	76.8	0.06	
Central Vietnam	32	14.0	8	7.1	40	11.7		
Southern Vietnam	19	8.3	15	13.3	34	10.0		
Abroad	5	2.2	0	0.0	5	1.5		
Age group								
Under 25	58	25.4	27	23.9	85	24.9	0.96	
25–34	68	29.8	34	30.1	102	29.9		
35–44	61	26.8	33	29.2	94	27.6		
Above 44	41	18.0	19	16.8	60	17.6		
Religion								
Yes	192	84.2	98	86.7	290	85.0	0.54	
No	36	15.8	15	13.3	51	15.0		
Marital status								
Single	82	36.0	45	39.8	127	37.2	0.39	
Living with spouse	139	61.0	67	59.3	206	60.4		
Others	7	3.1	1	0.9	8	2.4		
Family size								
1–2 people	41	18.0	23	20.4	64	18.8	0.40	
3–5 people	166	72.8	75	66.4	241	70.7		
Above 5 people	21	9.2	15	13.3	36	10.6		
Education level								
High school and below	44	19.3	31	27.4	75	22.0	0.13	
Undergraduate	131	57.5	53	46.9	184	54.0		
Postgraduate	53	23.3	29	25.7	82	24.1		
Occupation								
Health workers	47	20.6	19	16.8	66	19.4	0.22	
Professional educators	33	14.5	23	20.4	56	16.4		
White collar workers	44	19.3	27	23.9	71	20.8		
Students	55	24.1	29	25.7	84	24.6		
Others	49	21.5	15	13.3	64	18.8		
Occupation status								
Tenure	74	32.5	40	35.4	114	33.4	0.76	
Unlimited term fulltime contract	43	18.9	23	20.4	66	19.4		
Limited term fulltime contract	24	10.5	10	8.9	34	10.0		
Farmers/Students/Homemakers/Unemployed/Retired	69	30.3	35	31.0	104	30.5		
Others	18	7.9	5	4.4	23	6.7		
	Mean	SD	Mean	SD	Mean	SD	*p*-value	
Number of children	1.2	1.0	1.0	1.0	1.1	1.0	0.21	
Number of children aged above 15	0.3	0.6	0.3	0.8	0.3	0.7	0.92	
Age	33.4	10.3	34.2	11.8	33.7	10.8	0.85	

**Table 2:** Demand for health information about COVID-19.

Characteristics	*n*	%	Updated Information on Disease and Treatment	Transmission Mechanism and Specific Notices	Epidemiology of Symptoms, Treatment, and Prevention
Updated news about pandemic	259	76.0	0.84		
Information on disease symptoms	218	63.9			0.84
Updated news on outbreak	208	61.0	0.78		
Notices on how to prevent transmission	199	58.4			0.83
Notices for those who need specific information	199	58.4		0.50	
Number of new cases and their location	188	55.1	0.71		
The mechanism of transmission	185	54.3		0.62	
Availability and effectiveness of drugs or vaccines	183	53.7	0.55		
How other countries respond to the pandemic	172	50.4		0.62	
Notices on treatments	170	49.9			0.60
Notices when traveling	63	18.5		0.78	
Cronbach’s alpha			0.79	0.75	0.77
Mean			0.6	0.5	0.6
SD			0.4	0.4	0.4
Ceiling effect			38.1	15.8	39.9
Floor effect			16.4	24.6	24.3

**Table 3:** Factors associated with demand for health information on COVID-19.

Characteristics	Updated Information on Disease and Treatment		Transmission Mechanism and Specific Notices		Epidemiology of Symptoms, Treatment, and Prevention	
	Coef.	95% CI	Coef.	95% CI	Coef.	95% CI
Current living location (vs. Northern Vietnam)						
Central Vietnam					0.37 *	0.01; 0.73
Southern Vietnam			−0.20	−0.41; 0.00		
Abroad					0.67	−0.32; 1.66
Age group (vs. Under 25)						
35–44	0.85	0.68; 1.07	−0.20 *	−0.35; −0.05	−0.45 *	−0.73; −0.18
Above 44	1.09	0.88; 1.35				
Education level (vs. High school and below)						
Undergraduate	1.18	0.98; 1.42	0.15 *	0.02; 0.28	0.14	−0.19; 0.46
Postgraduate					−0.53 *	−0.90; −0.16
Occupation (vs. Health workers)						
Professional educators					0.63 *	0.27; 0.99
White-collar workers			−0.15	−0.31; 0.01	0.44 *	0.11; 0.78
Others					0.58 *	0.24; 0.93
Occupation status (vs. tenure)						
Limited term full-time contract	0.81	0.60; 1.09	0.23 *	0.01; 0.45	0.37	−0.05; 0.78
Others			−0.28 *	−0.53; −0.03	−0.56 *	−1.03; −0.10

* *p* < 0.05.
